# Applicability of the Disruptions in Surgery Index in the Cardiovascular Management Scenarios - A Marker for Developing Functionally Efficient Teams

**DOI:** 10.21470/1678-9741-2020-0685

**Published:** 2021

**Authors:** Vinicius Nina, Augusto Gonçalves Mendes, Nick Sevdalis, Aubyn Marath, Omar Vilca Mejia, Carlos Manuel A. Brandão, Rosangela Monteiro, Vinícius Giuliano Mendes, Fabio B Jatene

**Affiliations:** 1 Department of Medicine I, Universidade Federal do Maranhão, São Luís, Maranhão, Brazil.; 2 Health Service & Population Research, King's College London, London, London, United Kingdom of Great Britain and Northern Ireland.; 3 CardioStart International, Tampa, Florida, United States of America.; 4 Department of Cardiovascular Surgery, Instituto do Coração, Hospital das Clínicas, Faculdade de Medicina, Universidade de São Paulo, São Paulo, São Paulo, Brazil.; 5 Hospital do Câncer Tarquínio Lopes Filho, São Luís, Maranhão, Brazil.; 6 Department of Cardiac Surgery, Instituto do Coração, Faculdade de Medicina, Universidade de São Paulo, São Paulo, São Paulo, Brazil.

**Keywords:** Patient Safety, Cardiovascular Surgery, Problem Behavior, Perception, Self-Concept, Surgeons, Communication

## Abstract

**Introduction:**

To support the development of practices and guidelines that might help to reduce adverse events related to human factors, we aimed to study the response and perception by members of a cardiovascular surgery team of various error-driven or adverse features that might arise in the operating room (OR).

**Methods:**

A previously validated Disruptions in Surgery Index (DiSI) questionnaire was completed by individuals working together in a cardiovascular surgical unit. Results were submitted to reliability analysis by calculating the Cronbach’s alpha coefficient. Non-parametric Kruskal-Wallis test and Dunn’s post-test were performed to estimate differences in perceptions of adverse events or outcomes between the groups (surgeons, nurses, anesthesiologists, and technicians). *P*<0.05 was considered statistically significant.

**Results:**

Cronbach’s alpha reliability coefficients showed consistency within the recommended range for all disruption types assessed in DiSI: an individual’s skill (0.85), OR environment (0.88), communication (0.81), situational awareness (0.92), patient-related disruption (0.89), team cohesion (0.83), and organizational disruption (0.83). Nurses (27.4%) demonstrated significantly higher perception of disruptions than surgeons (25.4%), anesthetists (23.3%), and technicians (23.0%) (*P*=0.005). Study participants were more observant of their colleagues’ disruptive behaviors than their own (*P*=0.0001).

**Conclusion:**

Our results revealed that there is a tendency among participants to hold a positive self-perception position. DiSI appears to be a reliable and useful tool to assess surgical disruptions in cardiovascular OR teams, identifying negative features that might imperil teamwork and safety in the OR. And human factors training interventions are available to develop team skills and improve safety and efficiency in the cardiovascular OR.

**Table:** 

Abbreviations, acronyms & symbols	
DiSI	= Disruptions in Surgery Index
OR	= Operating room
UK	= United Kingdom

## INTRODUCTION

Historically, surgical outcomes are often attributed to the surgeon’s skill and the patient’s medical condition. Measurable elements such as teamwork, communication, physical environment, types of technologies, organizational factors, and workload are less well documented but may be crucial or contribute significantly to surgical performance and, ultimately, to the desired clinical results^(1)^.

Sevdalis et al.^(2)^ showed that the interaction between the members of a surgical team as well as the interaction with the surgical environment are determinants that merit careful analysis and a better understanding.

Disruptive elements can be defined as any event capable of compromising an individual’s ability to complete a task. The surgical environment imposes a high level of cognitive demand. Additionally, there are many elements capable of introducing disturbances to the perioperative workflow, such as inappropriate use of physical space, excessive entry and exit by operating room (OR) staff, electronic interference from beepers, and telephone calls during a case^(3)^,^(4)^. The most serious disrupting events, however, relate to equipment failures in the OR^(5)^,^(6)^.

Interruptions related to communication or background chatter can also negatively impact patient safety as they can become intrusive or distracting and may potentially compromise the assessment of essential details pertinent to the patient’s clinical condition^(7)^. Antoniadis et al.^(3)^ found that such interruptions tend to occur more frequently at the beginning of procedures. In 2017, Cohen et al.^(8)^ examined the first 50 hours of cardiovascular operations and demonstrated that 4 hours were wasted by anesthesiologists to resolve breaks in the surgical flow. In addition, resident physicians appeared to be more prone to error when compared to experienced surgeons^(9)^.

Individuals with different specialist backgrounds who share the perioperative management care when treating a patient may experience considerable professional challenges, which may provoke tensions in the interactions with each other when working in a perioperative environment, especially under high pressure and demand^(10)^-^(12)^.

Sevdalis et al.^(13)^ found that the surgical team and its team members can also be distracted by case-irrelevant communications, which have the potential to interfere with detailed technical demands of the surgery for which effective coordinated communication must be implemented to reduce such interference.

Based on our own experience and those of others^(14)^-^(16)^, we were concerned by the frequency and variety of potential disruptions occurring during cardiovascular operations, and their possible detrimental effect on safety and patient outcomes. We, therefore, designed a study that aims to evaluate the perceptions by members of a local cardiovascular surgery OR team of the various disruptive elements that might occur in the OR. We used a previously validated instrument to assess such disruptions, namely the Disruptions in Surgery Index (DiSI), which we have started to revalidate through cross-cultural translation and adaptation into Portuguese in a previous study^(16)^. Thus, the secondary aim of this study was to provide further validation evidence for the use of DiSI in Brazilian cardiovascular ORs.

## METHODS

### Ethics

In accordance with Resolution 466/12 of the National Health Council of Brazil, this study was submitted to the appreciation of the Ethics and Institutional Research Committee, having been approved under substantiated statement number 807780. All participants who agreed to participate in the research signed the authorized Informed Consent Form (or ICF).

### Study Design

An observational, analytical-descriptive cross-sectional study was carried out between January 2016 and December 2018 at the OR of a University Hospital (a tertiary care center for cardiovascular surgery) in the Northeast of Brazil.

Through convenience sampling, we selected those OR staff members who regularly participate directly in cardiovascular surgery management: surgeons, anesthesiologists, nurses, and technicians. Sixty potentially eligible individuals were subsequently identified. Fifty-five people participated in the study and completed the DiSI questionnaire; 12 were excluded for having returned the questionnaire incomplete. The final sample consisted of 43 participants who responded to the questionnaires in full.

### Description of the Tool

**Fig. 1:**
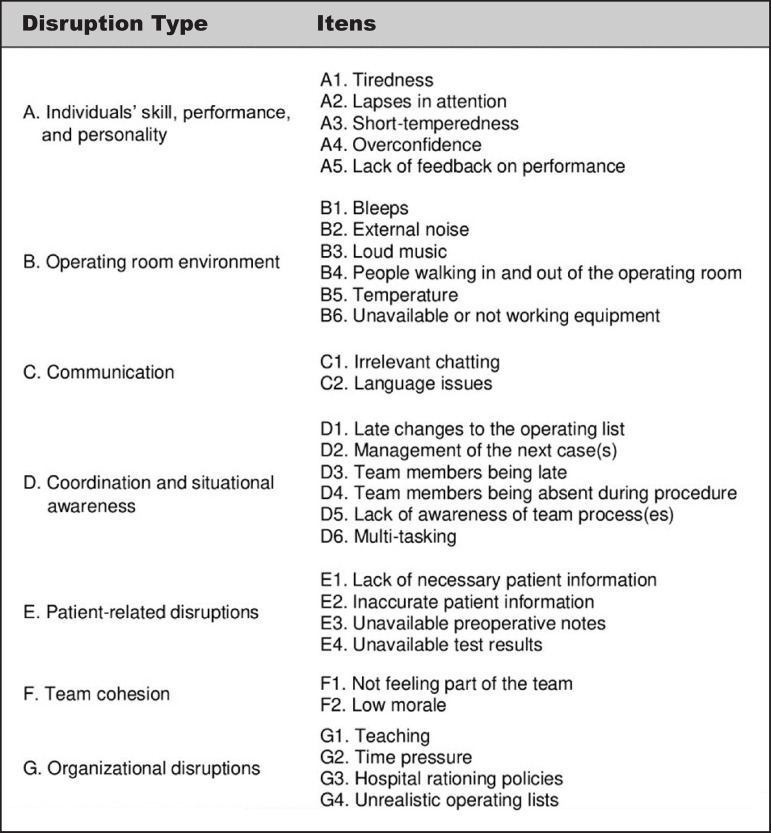
Disruptions in Surgery Index dimensions

DiSI is a tool in the form of a questionnaire developed from observational studies carried out in ORs in the United Kingdom (UK). DiSI assesses disruptive events in the OR, which are structured into seven main categories, each of them subdivided into questions that should be answered by perioperative team members (Figure 1)^(2)^. The categories of the tool are as follows:

- **Individual skill, performance, and personality**: examines individual clinicians’ performance and personality.
- **OR environment**: the environmental conditions of an OR and the distractions caused by bleeps, phone calls, unavailable equipment, door openings, etc.
- **Communication**: this examines distracting communication exchanges caused by irrelevant conversations as well as language barriers.
- **Coordination and situational awareness**: the conduct of the members of the surgical team of being focused on patient safety, their commitment and responsibility with schedules and learning from errors.
- **Patient-related disruptions**: the surgical team’s access to full and accurate information on the surgical patient.
- **Team cohesion**: individual team-members’ perceptions relating to their feeling part of and identifying with the team.
- **Organizational disruptions**: issues that affect working in an OR, delivery of surgical services and teaching occurring concurrently, and time pressures (staffing levels, waiting lists, etc.)^(2)^.

For each speciﬁc disruption type, the participants of the study provided the following measures:

- How often, on average, they observe a speciﬁc disruption in the OR (percentage scale: 0 percent = disruption is never observed; 100 percent = disruption is always present).
- How much each disruption contributes to potential error (10-point scale: 0 = not at all; 9 = extremely).
- How much each disruption obstructs the achievement of the goals of the procedure (10-point scale: 0 = not at all; 9 = extremely).

Participants provided these ratings twice: once for themselves and once again for their colleagues in the OR resulting in six measures collected per disruption.

### Statistical Analyses

Stata software version 14 (Statacorp LP, College Station, Texas, United States of America) was used for data analysis. Descriptive statistics was performed by calculating and tabulating means and standard deviations. For the reliability analysis, Cronbach’s alpha coefficient was calculated to estimate internal consistency. The value considered minimally acceptable was 0.7 or higher, as per established guidance for self-report measures^(17)^-^(19)^.

For the purposes of analysis, the data were grouped into two types of variables - dependent and independent. The following were considered as dependent variables: a) estimated frequency of disruptions, b) contribution to error, and c) obstruction of goals of the procedure. Target (self-perception and evaluation of colleagues), occupation (surgeons, nurses, technicians, and anesthesiologists), and type of disruption (A, B, C, D, E, F, G - as per DiSI classification) were considered independent variables.

The dependent variables were submitted to normality tests demonstrating that they did not follow a Gaussian distribution. To estimate participants’ perception of distinct disruptions, the data were submitted to the non-parametric Kruskal-Wallis test and then to Dunn’s post-hoc test.

## RESULTS

### Descriptive Analyses

A total of 7,482 responses provided by 43 individuals whose profile was composed of surgeons (20.9%), nurses (23.3%), technicians (37.2%), and anesthesiologists (18.6%) were recorded for analysis. Results of descriptive statistics, with calculation of the means and standard deviations of self-judgments and the judgments of colleagues, are described in Table 1.

**Table 1:** Descriptive analyses of self-judgments and judgments for others (n=43).

Disruption type	Item focus	Judgments for self*	Judgments for others*
A. Individuals' skill, performance, and personality	Frequency	19.8% (20.3%)	29.2% (21.8%)
	Contribution to error	3.56 (2.60)	4.61 (2.52)
	Obstruction of goals	3.29 (2.53)	4.24 (2.66)
B. Operating room environment	Frequency	21.7% (24.08%)	29.6% (25.03%)
	Contribution to error	2.71 (2.55)	3.85 (2.66)
	Obstruction of goals	2.79 (2.59)	3.51 (2.63)
C. Communication	Frequency	18.15% (16.18%)	25.24% (20.71%)
	Contribution to error	3.05 (2.47)	4.08 (2.56)
	Obstruction of goals	3.06 (2.59)	3.79 (2.83)
D. Coordination and situational awareness	Frequency	23.94% (24.83%)	31.6% (25.08%)
	Contribution to error	3.15 (2.61)	4.21 (2.55)
	Obstruction of goals	3.40 (2.65)	4.23 (2.65)
E. Patient-related disruptions	Frequency	26.08% (24.75%)	32.3% (28.24%)
	Contribution to error	3.89 (2.85)	4.43 (2.73)
	Obstruction of goals	3.93 (2.64)	4.7 (2.77)
F. Team cohesion	Frequency	9.37% (14.6%)	13.9% (16.5%)
	Contribution to error	1.9 (2.32)	2.72 (2.64)
	Obstruction of goals	2.15 (2.62)	2.92 (2.65)
G. Organizational disruptions	Frequency	19.24% (20.59%)	26.09% (24.29%)
	Contribution to error	2.51 (2.52)	3.44 (2.56)
	Obstruction of goals	2.8 (2.48)	3.66 (2.64)

* Means (standard deviations) of disruption scores across disruption types and self versus others focus

### Reliability Analyses

The results of the reliability analyses for each type of disruption are summarized in Table 2. It is observed that item D (Coordination and situational awareness) presented the highest coefficient, with a value > 0.9. The other items obtained coefficients between 0.7 and 0.9, indicating acceptable internal consistency.

**Table 2:** Reliability analyses of the disruption type (n=43).

Disruption type	Cronbach's alpha coefﬁcients
A. Individuals' skill, performance, and personality	0.85
B. Operating room environment	0.88
C. Communication	0.81
D. Coordination and situational awareness	0.92
E. Patient-related disruptions	0.89
F. Team cohesion	0.83
G. Organizational disruptions	0.83

Detailed reliability coefficients based on both self-judgments and judgments of colleagues and analyzed regarding estimated frequency, contribution to error, and obstruction of goals are summarized in Table 3.

**Table 3:** Reliability analyses of self-judgments and judgments for others* (n= 43).

Disruption type	Item focus	Judgments for self	Judgments for others
A. Individuals' skill, performance, and personality	Frequency	0.60	0.77
	Contribution to error	0.83	0.85
	Obstruction of goals	0.92	0.92
B. Operating room environment	Frequency	0.78	0.84
	Contribution to error	0.87	0.88
	Obstruction of goals	0.86	0.90
C. Communication	Frequency	0.71	0.67
	Contribution to error	0.85	0.85
	Obstruction of goals	0.86	0.86
D. Coordination and situational awareness	Frequency	0.90	0.92
	Contribution to error	0.89	0.88
	Obstruction of goals	0.91	0.89
E. Patient-related disruptions	Frequency	0.90	0.96
	Contribution to error	0.92	0.92
	Obstruction of goals	0.91	0.91
F. Team cohesion	Frequency	0.86	0.92
	Contribution to error	0.90	0.88
	Obstruction of goals	0.92	0.90
G. Organizational disruptions	Frequency	0.72	0.78
	Contribution to error	0.75	0.77
	Obstruction of goals	0.70	0.80

* Cronbach's alpha coefﬁcients

### Impact of Disruptions

#### Frequency

Participants judged that the various types of disruption occur with different frequencies (*P*=0.0001). The results also showed statistically lower average for self-perception (21.02%) of disruption than perception for colleagues (28.4%) (*P*=0.0001).

Among the four staff groups, nurses (27.4%) demonstrated significantly higher perception of disruptions than surgeons (25.4%), anesthesiologists (23.3%), and technicians (23.0%) (*P*=0.0052). This difference remained significant in the post-test between technicians and other specialties: surgeons (*P*=0.0024), nurses (*P* = 0.0021), and anesthesiologists (*P*=0.0043), as shown in Table 4.

**Table 4:** Frequency of disruptions according to the occupation of participants* (n = 43).

Occupation	Surgeons	Nurses	Technicians
Nurses	*P*=0.4816	---	---
Technicians	*P*=0.0024	*P*=0.0021	---
Anesthesiologists	*P*=0.4634	*P*=0.4804	*P*=0.0043

* Dunn's test

#### Contribution to Error

Regarding the contribution to error, on a scale of 0 to 9 points, participants were more observant of their colleagues (mean = 4.02) disruptive behavior than their own (mean = 3.05) (*P*=0.0001).

There was also significance between the categories of disruptions, demonstrating that they contributed distinctly to the occurrence of errors (*P*=0.0001). Among the four staff groups, significantly different mean values of contribution to error were observed: surgeons (3.72), nurses (3.84), technicians (3.38), and anesthesiologists (3.24) (*P*=0.0014).

#### Obstruction of Goals

**Fig. 2:**
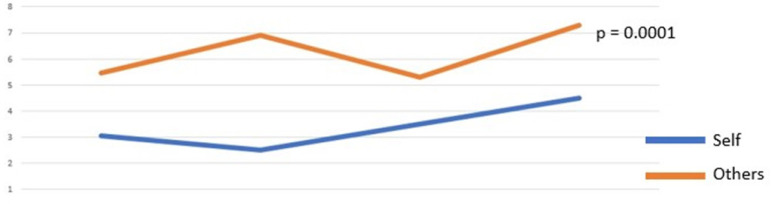
Obstruction of goals according to self-judgments and judgments for others.

As for the obstruction of goals, on a scale from 0 to 9, a similar behavior was observed with participants being more observant of their colleagues (mean = 3.95) incompleteness of tasks than their own (mean = 3.13) (*P*=0.0001) (Figure 2).

Nurses (mean = 3.79) were more observant of the impact of disruptions resulting in obstruction of goals than surgeons (3.70), technicians (3.49), and anesthesiologists (3.20) (*P*=0.0068).

## DISCUSSION

The results of the present study demonstrated that DiSI is a tool that can reliably capture the perceptions of members of a cardiovascular surgery team of various error-driven or *de novo* adverse events that might arise in the OR during clinical care of patients. Further, participants of this study judged disruptions occur to and affect their colleagues more than themselves. Both findings replicate the original UK data, which also indicated that DiSI could be used reliably and also a significant self-other gap in perceptions of how much a disruptive event impacts on staff members in the OR.

The reliability analysis by Cronbach’s alpha represents one of the main resources used to evaluate a given construct. We chose to use this evaluative tool to determine if each type of disruption evaluated by DiSI had the same latent trait, and if there was any redundancy or discrepancy among the selected questions. Most of the obtained measures in this study showed satisfactory reliability, although disruption type D (Coordination and situational awareness) showed a result (0.92) that could suggest removing some of the items from this category in future applications (as they may be redundant)^(20)^-^(22)^.

Our results also demonstrated a positive self-perception in our sample of OR professionals, who judged that disruptive events potentially impact on their colleagues significantly more than they impact on themselves. This may be a reflection of individual commitment to teamwork, especially when taking into account that there may be no hierarchic alignment between members about the tasks to be accomplished by each of them in a particular case^(23)^. The results, however, may also reflect a “rosy” self-perception, such that the staff in our study may underestimate the potential impact of disruptive event on the safety of their own work. Further study is required to identify the cause of such positive self-perceptions and their potential impact on surgical safety.

Several issues can cause interruption of the standard functioning of a procedure. Distractions, for example, especially those related to equipment problems, as judged by participants of this study, are associated with poor overall teamwork and higher stress levels. And, in this scenario, nurses seem to be more aware of such disruption given that equipment preparation and management falls directly within their professional role and responsibilities^(5)^. Other frequent sources of distraction in the OR were conversations, telephones, people walking in and out, and radios. These findings are in accordance with Mentis et al.^(6)^ who showed that the most serious distractions were those related to defective equipment, the procedure itself, and futile and cellular conversations.

The mismatch between frequency and severity of events (mainly distractions) do have different impacts within the OR. We also demonstrated similar features in the present study.

Team cohesion and collaboration are fundamental for adequate and consistent standards of surgical flow. Human elements that compromise teamwork were identified in this study, which were illustrated by the way individuals interpreted the DiSI questionnaire: they showed a tendency to blame their colleagues. An Italian study, which evaluated 42 adverse events, showed that 31 of them were related to poor teamwork, with inaccurate verbal and written communication in addition to inadequate transmission of patient information^(15)^. In a 2018 literature review, the authors demonstrated that one in 10 patients is affected by some type of adverse event, with one in 14 of those events resulting in death; half of these were viewed as preventable^(24)^.

To mitigate such adverse events, features of crew resource management derived from aviation can be readily applied to healthcare settings such as the OR, and these include peer monitoring, briefings, defining operating procedures and standards, recognition of fatigue as a factor in performance, blame-free reporting culture, use of checklists, and application of the principle of a “sterile cockpit”, which essentially refers to an environment free of unnecessary distractions preserving patient safety^(25)^.

When analyzing the perception of team members in greater detail, our study revealed that different interpretations were observed among those from different sub-specialist training backgrounds. This may be explained by the distinct role played by those professionals in each step of the procedure incurring in different perception among them regarding the frequency, magnitude, and severity of the disruption of the surgical flow and, ultimately, contribution to error^(3)^,^(5)^-^(9)^. However, simple measures can break those sociotechnical barriers and improve team’s cohesion and assertiveness, such as use of first names in interactions, direct eye contact, introducing each other, using non-judgmental words, and putting safety before self-esteem. Adoption of those measures in the OR may be particularly helpful in both encouraging members in the team to stand back and appraise procedures and also to encourage mutual respect and team bonding between the members.

One interesting aspect of the study is that it replicates the results of DiSI studies carried out several years ago with OR staff in the UK^(2)^. In the British data, DiSI was also found to be reliable in assessing perceptions of disruptive events, the different types of events were scored similarly, and lastly the self-other difference in perception of how much a disruption impacts on one’s performance was also found. These similarities suggest that OR safety culture and perceptions may have elements shared across different national cultures. These may be the result, for example, of the professional acculturation into the surgical and other perioperative professions, which is possibly similar across different countries. Further cross-national studies are required to test this hypothesis.

### Limitations

The limitations in this study include convenience sampling, small sample size, data based on self-report, single institution study, and inevitable bias from team members working together in some cases for years.

Some measurement bias may have occurred among participants as each answered the questionnaire at times and places they chose and perceptions of frequency and seriousness may have been different among the participants^(2)^,^(5)^. Strengths of the study include the fact that the number of participants was similar to those already published in previous DiSI studies, which makes our results relatively comparable^(2)^, and the conduct of the study amongst a group of experienced professional used to working together, which means the study data are useful for further team skills development of our participants. Future studies will require larger sample size and more surgical centres to be involved, to expand the applicability and generalizability of the findings.

## CONCLUSION

We carried out this study seek in Brazil to help educate surgical teams about the source of disruptions that might threaten safe surgical routines and also to gain a better appreciation of the dynamics and perceptive features that may arise among colleagues working together. It is hoped that our findings will nurture the development of practices capable of reducing adverse events related to human factors and improve surgical outcomes that follow internationally accepted standards and guidelines.

DiSI appears to be a reliable and useful tool to assess surgical disruptions in the cardiovascular scenario. Our results revealed that there is a tendency among participants to hold a positive self-perception position; the research tool also helped identify colleagues who initiate or contribute to negative or disruptive behavior within the OR. We believe these tools and measures may help nurture positive team dynamics, a clearer understanding of errors in perioperative management, and identify negative features that might imperil a program’s function and growth.

**Table:** 

Authors' roles & responsibilities	
VN	Substantial contributions to the conception of the work; and the acquisition and analysis of data for the work; drafting the work; final approval of the version to be published
AGM	Substantial contributions to the conception of the work; and the acquisition and analysis of data for the work; drafting the work; final approval of the version to be published
NS	Substantial contributions to the conception of the work; and the acquisition and interpretation of data for the work; final approval of the version to be published
AM	Substantial contributions to the acquisition, analysis, and interpretation of data for the work; drafting the work; final approval of the version to be published
OVM	Substantial contributions to the acquisition and interpretation of data for the work; final approval of the version to be published
CMAB	Substantial contributions to the acquisition and interpretation of data for the work; final approval of the version to be published
RM	Substantial contributions to the acquisition and interpretation of data for the work; final approval of the version to be published
VGM	Substantial contributions to the acquisition, analysis, and interpretation of data for the work; final approval of the version to be published
FBJ	Substantial contributions to the acquisition and interpretation of data for the work; final approval of the version to be published

## References

[1] ElBardissi AW, Sundt TM (2012). Human factors and operating room safety. Surg Clin North Am.

[2] Sevdalis N, Forrest D, Undre S, Darzi A, Vincent C (2008). Annoyances, disruptions, and interruptions in surgery the
disruptions in surgery index (DiSI). World J Surg.

[3] Antoniadis S, Passauer-Baierl S, Baschnegger H, Weigl M (2014). Identification and interference of intraoperative distractions
and interruptions in operating rooms. J Surg Res.

[4] Palmer 2nd G, Abernathy 3rd JH, Swinton G, Allison D, Greenstein J, Shappell S (2013). Realizing improved patient care through human-centered operating
room design a human factors methodology for observing flow disruptions in
the cardiothoracic operating room. Anesthesiology.

[5] Wheelock A, Suliman A, Wharton R, Babu ED, Hull L, Vincent C (2015). The impact of operating room distractions on stress, workload,
and teamwork. Ann Surg.

[6] Mentis HM, Chellali A, Manser K, Cao CG, Schwaitzberg SD (2016). A systematic review of the effect of distraction on surgeon
performance directions for operating room policy and surgical
training. Surg Endosc.

[7] Sevdalis N, Undre S, McDermott J, Giddie J, Diner L, Smith G (2014). Impact of intraoperative distractions on patient safety a
prospective descriptive study using validated instruments. World J Surg.

[8] Boquet A, Cohen T, Diljohn F, Cabrera J, Reeves S, Shappell S. (2017). J Patient Saf.

[9] Persoon MC, Broos HJ, Witjes JA, Hendrikx AJ, Scherpbier AJ (2011). The effect of distractions in the operating room during
endourological procedures. Surg Endosc.

[10] Katz JD (2007). Conflict and its resolution in the operating room. J Clin Anesth.

[11] Attri JP, Sandhu GK, Mohan B, Bala N, Sandhu KS, Bansal L (2015). Conflicts in operating room focus on causes and
resolution. Saudi J Anaesth.

[12] Hall J, Tobias JD (2016). Operating room conflict resolution time to figure it
out. Saudi J Anaesth.

[13] Sevdalis N, Healey AN, Vincent CA (2007). Distracting communications in the operating
theatre. J Eval Clin Pract.

[14] Albolino S, Tartaglia R, Bellandi T, Bianchini E, Fabbro G, Forni S (2017). Variability of adverse events in the public health-care service
of the Tuscany region. Intern Emerg Med.

[15] Bellandi T, Tartaglia R, Forni S, D&apos;Arienzo S, Tulli G (2017). Adverse events in cardiac surgery, a mixed methods retrospective
study in an Italian teaching hospital. J Eval Clin Pract.

[16] Nina VJDS, Jatene FB, Sevdalis N, Mejía OAV, Brandão CMA, Monteiro R (2017). Pre-validation study of the Brazilian version of the disruptions
in surgery index (DiSI) as a safety tool in cardiothoracic
surgery. Braz J Cardiovasc Surg.

[17] Streiner DL (2003). Being inconsistent about consistency when coefficient alpha does
and doesn&apos;t matter. J Pers Assess.

[18] Brown TA (2014). Confirmatory factor analysis for applied research.

[19] Stacciarini TS, Pace AE (2017). Confirmatory factor analysis of the appraisal of self-care agency
scale - revised. Rev Lat Am Enfermagem.

[20] Helms JE, Henze KT, Sass TL, Mifsud VA (2006). Treating cronbach's alpha reliability coefficients as data in
counseling research. Couns Psychol.

[21] Cho E, Kim S (2015). Cronbach's coefficient alpha well known but poorly
understood. Organ Res Methods.

[22] Acock A. (2014). A Gentle Introduction to Stata.

[23] Nakarada-Kordic I, Weller JM, Webster CS, Cumin D, Frampton C, Boyd M (2016). Assessing the similarity of mental models of operating room team
members and implications for patient safety a prospective, replicated
study. BMC Med Educ.

[24] Schwendimann R, Blatter C, Dhaini S, Simon M, Ausserhofer D (2018). The occurrence, types, consequences and preventability of
in-hospital adverse events - a scoping review. BMC Health Serv Res.

[25] Kapur N, Parand A, Soukup T, Reader T, Sevdalis N (2015). Aviation and healthcare a comparative review with implications
for patient safety. JRSM Open.

